# Effectiveness and safety of a glutaraldehyde-modified, L-tyrosine-adsorbed and monophosphoryl lipid A-Adjuvanted allergen immunotherapy in patients with allergic asthma sensitized to olive pollen: A retrospective, controlled real-world study^☆^

**DOI:** 10.1016/j.waojou.2020.100487

**Published:** 2020-12-18

**Authors:** José Fernando Florido-López, Carmen Andreu-Balaguer, Carmelo Escudero, Marta Seoane-Rodríguez, Mercedes Hernández, Luis Ángel Navarro-Seisdedos, Miguel Torrecillas-Toro, Mónica Anton-Girones, Leticia Herrero-Lifona, Dorimar Brugaletta, Jesús Macías, Rafael Pineda, Maria Ángeles Lara, Julián López-Caballero, Maria José Rojas

**Affiliations:** a Hospital Clínico Universitario (Parque Tecnológico de La Salud). Granada, Spain; b Hospital Comarcal de Orihuela. Alicante, Spain; c Department of Allergy, Hospital Infantil Universitario Niño Jesús, IIS-P, FibHNJ. Madrid, Spain; d Hospital Infanta Elena and Hospital de Valdemoro. Madrid, Spain; e Hospital Nisa Sevilla Aljarafe. Sevilla, Spain; f Hospital Lluis Alcanyís, Xàtiva, Valencia, Spain; g Hospital Perpetuo Socorro – Albacete. Albacete, Spain; h Hospital Universitario de Vinalopó. Alicante, Spain; i Hospital Quirón Málaga. Málaga, Spain; j Hospital Arganda. Madrid, Spain; k Hospital Vithas Granada. Granada, Spain

**Keywords:** Allergen immunotherapy, Olive pollen, Allergic asthma, Allergic rhinitis, Subcutaneous immunotherapy, Microcrystalline tyrosine, Allergoid, Monophosphoryl lipid A, AAAAI, American Academy of Allergy, Asthma & Immunology, AIT, allergen immunotherapy, ARs, adverse reactions, ARIA, Allergic Rhinitis and its Impact on Asthma, EAACI, European Academy of Allergy and Clinical Immunology, GEMA, “Guía Española para el Manejo del Asma” (Spanish Guidelines for Asthma Management), GINA, Global Initiative for Asthma, LABAs, long-acting beta-2 agonists, LTRAs, leukotriene receptor antagonists, MCT, microcrystalline tyrosine, MPL, monophosphoryl lipid A, SABAs, short-acting beta-agonists, SCIT, subcutaneous immunotherapy

## Abstract

**Background:**

Allergy to olive pollen is one of the primary causes of allergic asthma in Spain. Even though allergen immunotherapy (AIT) has shown clinical benefits in patients sensitized to different allergens, studies in asthmatic patients sensitized to olive pollen are insufficient.

**Objective:**

To assess the effectiveness and safety of an ultra-short course of AIT with an L-tyrosine-adsorbed and monophosphoryl lipid A-adjuvanted olive pollen and olive/grass pollen extract (Pollinex Quattro®) in patients with allergic asthma in the real-world setting.

**Methods:**

Retrospective, controlled study including patients with asthma, with and without allergic rhinitis, caused by sensitization to olive pollen from 11 centers in Spain. Patients received out-of-season (October–March) treatment with AIT in addition to their pharmacological treatment (active group) or pharmacological treatment (control group). Effectiveness variables, including unscheduled visits to the healthcare center, emergency room admissions, symptoms of asthma and rhinitis (following GEMA and ARIA classifications, respectively), and use of medication to treat asthma and rhinitis during the subsequent pollen season were compared between treatment groups.

**Results:**

Of 131 study patients, 42 were treated with their usual asthma medication (control group) and 89 were treated with AIT (active group), either Pollinex Quattro® 100% olive pollen (n = 43, 48.3%) or 50% olive pollen/50% grass pollen (n = 46, 51.7%). Patients’ demographic and clinical characteristics were similar between groups. The mean (SD) number of unscheduled visits to a healthcare center and emergency room admissions due to allergy symptoms was 2.19 (1.40) and 0.43 (0.63) in the control group, and 1.09 (1.25) and 0.11 (0.51) in the active group (*P* = 0.001 and *P* = 0.006, respectively). Severity and control of asthma symptoms remained unchanged (*P* = 0.347 and *P* = 0.179, respectively), rhinitis type improved (*P* = 0.025), and severity remained unchanged in the active compared to the control group. The use of short-acting beta-agonists and inhaled corticosteroids to treat asthma symptoms decreased in the active vs. the control group (*P* = 0.001 and *P* = 0.031, respectively). Twelve (13.5%) and two (2.2%) patients in the active group experienced local adverse reactions (edema, swelling, erythema, hives, pruritus, and heat), and systemic adverse reactions (hypertensive crisis and low-grade fever) to AIT, respectively; none was serious.

**Conclusion:**

AIT with Pollinex Quattro® specific for olive pollen and olive/grass pollens resulted in reduced visits to the healthcare center and emergency room and the use of asthma medication during the pollen season, indicating that this treatment was safe and effective in treating asthma in patients sensitized to these pollens.

## Introduction

Asthma is a chronic inflammatory disorder of the airways with an estimated prevalence of 1%–18% in different countries, affecting 300 million people worldwide, and is the most common chronic disease in children.^1^^,^^2^ Allergic asthma, the most frequent form of asthma, is commonly associated with allergic rhinitis and usually caused by sensitization to pollen grains, which may trigger asthma exacerbations.^3^^,^^4^

Sensitization of patients to different pollens varies regionally, being allergy to olive pollen particularly prevalent in the Mediterranean area.^5^, ^6^, ^7^, ^8^ In Spain, sensitization to olive pollen is, after grass pollen, the second cause of allergic asthma, and polysensitization to both pollens is frequent.^9^^,^^10^ In different regions with different predominant pollens, in each pollen season, increased pollen counts and/or pollen allergen concentrations (i.e., pollen peaks) have been associated with increased burden of asthma.^11^^,^^12^ However, compared to other pollens, olive pollen has shown a greater allergenic potency and a stronger association with disease exacerbation in asthmatic patients.^13^ Consequently, in regions with extensive *Olea europeae* cultivars, such as the south and center of Spain, olive pollen counts and allergen concentrations reach very high levels during the pollination season, causing increased incidence of asthma exacerbations and associated admissions to the emergency room, overloading the primary healthcare system and increasing direct and indirect asthma costs.^6^^,^^8^^,^^13^^,^^14^

To control asthma symptoms and reduce the risk of exacerbations, patients with allergic asthma receive conventional pharmacological treatment, consisting mainly in inhaled corticosteroids and long-acting beta-2 agonists (LABAs). Even though these treatments provide temporary control of symptoms, they are unable to alter disease progression.^1^ Allergen immunotherapy (AIT), commonly used to treat allergic rhinitis, has the unique potential of altering the course of the disease, providing long-term benefits to sensitized patients. Based on recent clinical evidence in patients with allergic asthma, guidelines are increasingly including AIT as a treatment option for this condition.^1^^,^^15^ Among the different AITs available, Pollinex Quattro® consists of pollen extracts modified by treatment with glutaraldehyde into allergoids and associated with two adjuvants—microcrystalline tyrosine (MCT) and monophosphoryl lipid A (MPL)—allowing for a short treatment course of four out-of-season injections. Pollinex Quattro® has shown good safety and efficacy profiles in previous clinical trials assessing rhinitis symptoms and the use of medication to treat rhinitis in patients sensitized to grass/rye, *Parietaria*, and/or tree pollens, including birch, alder, and hazel.^16^, ^17^, ^18^, ^19^, ^20^, ^21^, ^22^

Even though Pollinex Quattro® is prescribed to treat asthma due to olive pollen allergy, evidence is limited, raising the need for additional clinically relevant data.^23^^,^^24^ In this retrospective controlled study, we assessed the effectiveness and safety of Pollinex Quattro® in two cohorts of patients with seasonal allergic asthma, with or without rhinitis, caused by olive and by olive and grass pollen, treated and not treated with AIT according to routine clinical practice.

## Material and methods

### Study design and population

This was an observational, retrospective, multicenter study including patients with seasonal allergic asthma, with or without rhinitis, caused by olive or grass/olive pollens. Patients aged 5–65 years treated at any of the 11 participating Spanish centers, were assigned to one of the two cohorts of the study according to treatment. Cohort 1, or active treatment group, included patients receiving, besides their usual control medication to treat their allergic disease, allergen immunotherapy (Pollinex Quattro®) for the first time, according to the routine clinical practice, between October 2016 and March 2017, or between October 2017 and March 2018. Cohort 2, or control treatment group, included patients who visited the allergist for the first time after the usual out-of-season period of AIT treatment (October–March) and, therefore, received conventional pharmacological treatment according to the routine clinical practice and served as controls. Patients with poor treatment adherence were excluded from the study. Data were obtained from medical records between September 2018 and July 2019. All included patients and legal representatives of patients <18 years signed a written informed consent before any information was recorded. The study was conducted in accordance with the Helsinki Declaration and the local Personal Data Protection Law; the study protocol was approved by the local Ethics Committee (CEIM/CEI Provincial de Granada, Spain).

### Study medication

Pollinex Quattro® is an injectable suspension for subcutaneous administration composed of purified allergen extracts of 100% olive pollen or 50% olive pollen/50% grass pollen, modified by treatment with glutaraldehyde into allergoids, adsorbed onto microcrystalline tyrosine (MCT) and adjuvanted with monophosphoryl lipid A (MPL) at 50 μg/mL. AIT composition was selected according to the routine practice considering cutaneous sensitization, determined using a skin prick-test, detection of specific IgE in serum, anamnesis, and exposure to different pollens according to patients’ region of residence. This AIT is administered outside of the pollen season in four injections (ultra-short regimen) of 1 mL each, containing increasingly higher doses of the allergen (300 SU, 800 SU, and two injections of 2000 SU).

### Endpoints and variables

The objectives of this study were to assess the effectiveness (main objective) and safety (secondary objective) of AIT with Pollinex Quattro® in the real-world setting. Regarding effectiveness, the primary endpoint of this study was the number of unscheduled visits to a physician or healthcare center due to allergic disease during the pollen season. Secondary endpoints included the number of visits to the emergency room due to allergy symptoms, classification of asthma (type and severity), and use of medication to treat asthma, measured during the pollen season (2017 or 2018). In patients with allergic rhinitis, the classification of rhinitis symptoms and the use of medication to treat allergic rhinitis were also considered. Asthma was classified according to the “Guía Española para el Manejo del Asma” (GEMA), the Spanish version of the Global Initiative for Asthma (GINA) classification,^1^^,^^25^ and rhinitis was classified according to Allergic Rhinitis and its Impact on Asthma (ARIA) guidelines.^26^^,^^27^ GEMA and GINA classify asthma in two dimensions that, together, result in four categories (from less to more severe): intermittent, mild persistent, moderate persistent, and severe persistent. Asthma control was additionally classified according to GEMA as poorly, partially, and well controlled.^25^ ARIA classifies allergic rhinitis in two different dimensions—frequency and intensity—which include two and three categories, respectively. Ranging from less to more severe, allergic rhinitis frequency is classified as intermittent and persistent, whereas allergic rhinitis intensity is classified as mild, moderate, and severe.^26^^,^^27^ Additional variables recorded were demographic information, including age and sex, clinical characteristics, including disease duration (asthma and rhinitis), allergen sensitization, and other allergic diseases, and treatment characteristics, including the date of AIT start and composition. For the secondary objective of this study (i.e., safety and tolerability), the number and severity of local and systemic adverse reactions during treatment were recorded.

### Statistical analysis

The sample size was calculated based on the hypothesis that the proportion of patients in the control treatment group who would likely visit a healthcare center due to allergic asthma symptoms during the pollen season, estimated to be 75%, would be reduced by at least 25% (i.e., to 56%) in the active treatment group. With a 2:1 study design, including one patient receiving conventional pharmacological treatment (control treatment) for every two patients receiving AIT (active treatment) (2n in the active group:1n in the control group), and considering that 10% of patients would be non-assessable, a sample of 165 and 85 patients in the active and control treatment groups, respectively, was deemed necessary to detect differences between the two groups with a power of 80% and a significance level of 5%.

All analyses were performed based on the sample of assessable patients who fulfilled the selection criteria and presented with data about immunotherapy. Categorical variables were described as frequencies and percentages, and quantitative variables as the mean and standard deviation (SD) and/or the median and range. Categorical variables were compared using the Chi-square test or the Fisher test for subgroups of patients; quantitative variables were compared using the Student's T-test and their non-parametric counterpart, the Mann–Whitney test. In addition to analyses using all assessable study patients, effectiveness variables were compared in patient groups based on the composition of Pollinex Quattro® (50% olive pollen/50% grass pollen vs. 100% olive pollen) and the period of treatment administration (October–December and January–March). The significance threshold for all bivariate analyses was set at a two-sided α = 0.05. All analyses were performed using the statistical package SPSS version 19.

## Results

### Demographic, clinical and treatment characteristics of study patients

Of the 134 patients recruited for this study, three were non-assessable due to lack of data, resulting in a study population of 131 patients, of which 93 (71%) were adults (>18 years), 23 (17.5%) were adolescents (12–17 years), and 15 (11.4%), were children (5–11 years). Of the 131 assessable patients, 89 were treated with AIT (active group) (mean [SD] age of 31.6 [16.6]) and 42 were treated with their usual asthma medication (control group) (mean [SD] age of 28.8 [14.3]) years. Patients’ demographic and clinical characteristics are summarized in Table 1 and were similar between treatment groups (*P*; not significant), with the exception of rhinitis diagnosis and sensitization to molds, with a significantly different prevalence between the control and active groups (*P* = 0.019 and *P* = 0.002, respectively). Regarding AIT composition, 43 (48.3%) and 46 (51.7%) patients were administered Pollinex Quattro® composed of 100% olive pollen and Pollinex Quattro® composed of 50% olive pollen/50% grass pollen, respectively. The period of administration of AIT was from January to March in 68.5% of patients, and from October to December in the remaining 31.5%.

**Table 1:** Demographic, clinical, and treatment characteristics of study patients according to treatment, n = 131.

	Active Group n = 89	Control Group n = 42
**Demographic Characteristics**		
Age, years, *n (%)*		
5–11 (children)	9 (10.1)	6 (14.3)
12–17 (adolescents)	17 (19.1)	6 (14.3)
≥18 (adults)	63 (70.8)	30 (71.4)
Sex, female:male ratio (%)	48.3:51.7	52.4:47.6
**Clinical Characteristics**		
Asthma duration (years), *mean (SD)*	7.1 (7.2)	5.4 (3.9)
Rhinitis diagnosis, *n (%)*	88 (98.8)	38 (90.5)
Other allergen sensitization, *n (%)*		
Other pollens	50 (56.2)	26 (61.9)
Grass	46 (51.7)	23 (54.8)
Cypress	18 (22.8)	11 (29.7)
Platanus	15 (19)	4 (10.8)
Parietaria	9 (11.4)	3 (8.1)
Artemisia	7 (8.9)	0 (0)
Salsola	19 (24.1)	13 (35.1)
Chenopodium	11 (13.9)	5 (13.5)
Plantain	3 (3.8)	0 (0)
Palm tree	2 (0.5)	0 (0)
Pinus	0 (0)	1 (2.7)
Birch	1 (1.3)	0 (0)
House dust mites	18 (20.2)	15 (35.7)
Molds	5 (5.6)	10 (23.8)
Animal dander	25 (28.1)	18 (42.9)
Pollen Sensitization Profile, *n (%)*^a^		
Olive only	22 (24.7)	9 (21.4)
Olive and grass	17 (19.1)	7 (16.7)
Olive and other pollens	11 (12.4)	5 (11.9)
Olive, grass and other pollens	29 (32.6)	16 (38.1)

a Total number of patients per group; data regarding sensitization to pollens other than olive and grass was unavailable for 10 and 5 patients in the active and control treatment groups, respectively

### Visits to the healthcare center and emergency services

**Fig. 1:**
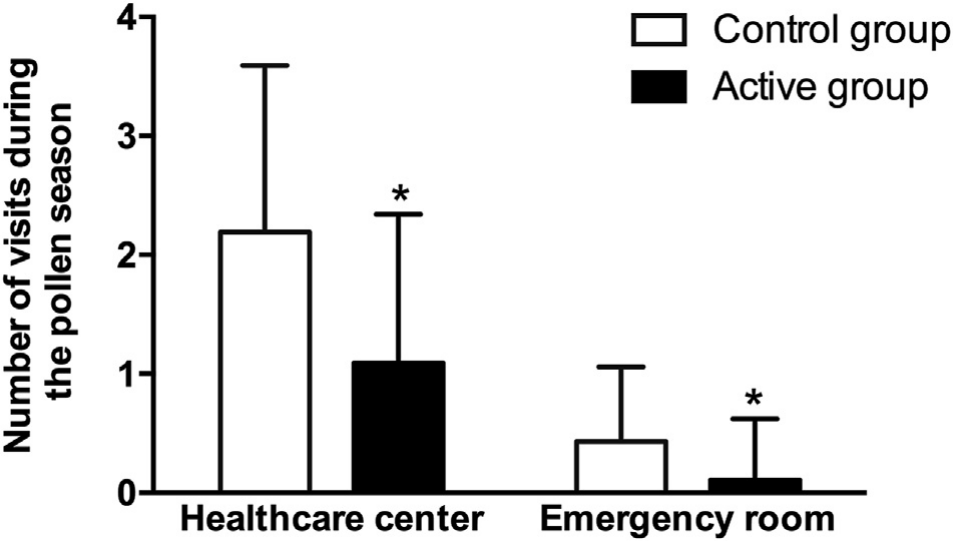
Mean number of unscheduled visits to the healthcare center (A) and to the emergency room (B) during the pollen season in patients in the control and active treatment groups. Columns and error bars represent the mean and standard deviation, respectively. ∗Student's T test, *P* < 0.05

Patients from the active and control groups experienced a mean (SD) of 1.09 (1.25) and 2.19 (1.40) (*P* = 0.001) unscheduled visits to a healthcare center and a mean (SD) of 0.11 (0.51) and 0.43 (0.63) (*P* = 0.006) emergency room admissions due to allergy symptoms during the pollen season, respectively (Fig. 1). Forty (95.2%) and 15 (35.7%) patients in the control group, and 56 (62.9%) and six (6.7%) patients in the active group visited the healthcare center and emergency room, respectively, during the pollen season (Chi-square test *P* < 0.001 for both comparisons). Consequently, the probability of visiting the healthcare center or emergency room was lower for patients in the active group compared to patients in the control group; healthcare center visits (OR = 0.08; 95% CI 0.02–0.37); emergency room visits (OR = 0.13; 95% CI 0.05–0.37).

### Symptoms of allergic asthma and rhinitis

**Fig. 2:**
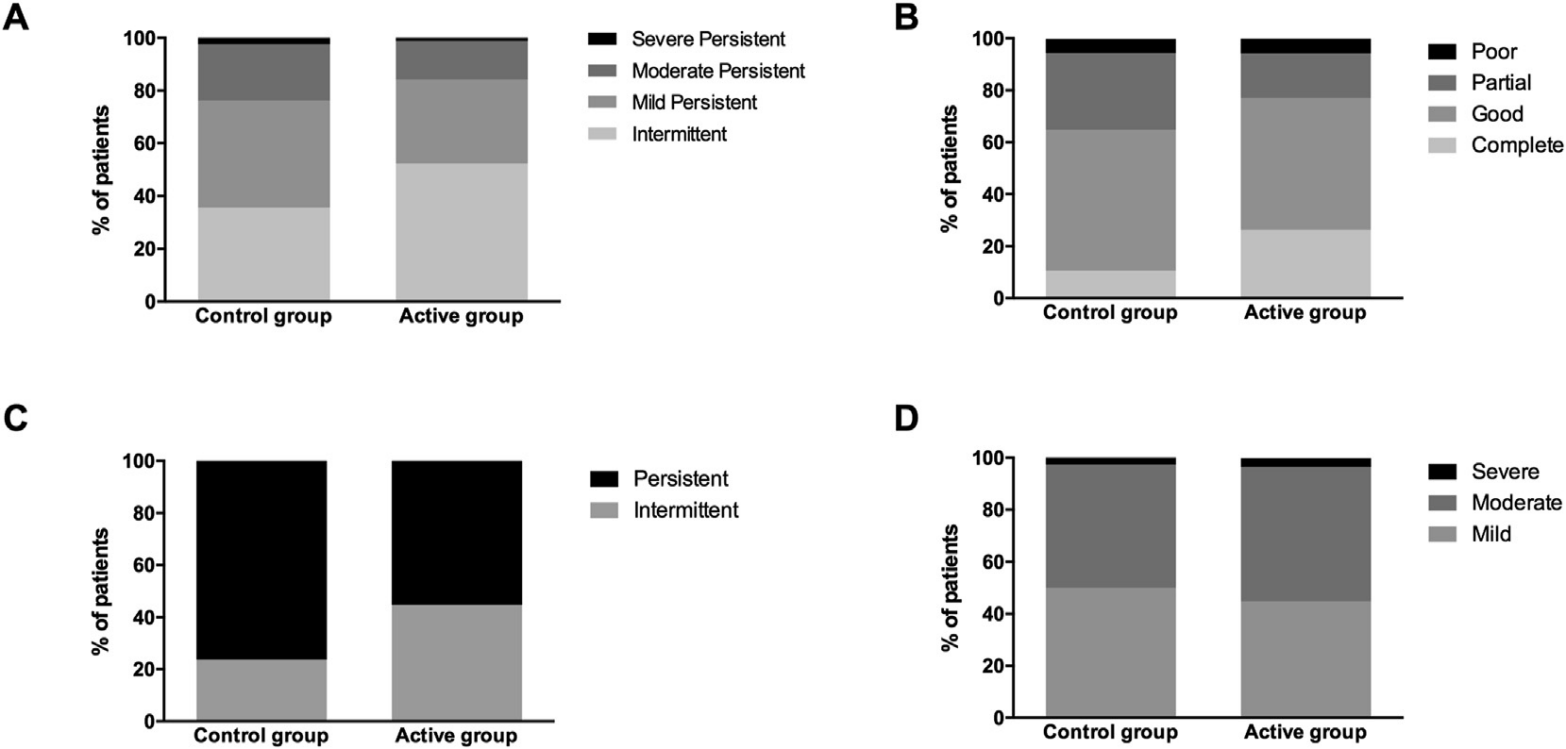
Distribution of patients in the control and active treatment groups according to asthma symptom severity (A) and control (B), rhinitis type (C), and rhinitis severity (D), according to the ARIA and GEMA classifications for rhinitis and asthma, respectively. The percentage of patients in each category is shown in the Y-axis. Chi-square test, *P* = 0.347, *P* = 0.179, *P* = 0.025, and *P* = 0.858, for A, B, C and D, respectively

The severity and control of asthma symptoms, according to the GEMA classification, was similar in active and control treatment groups (*P* = 0.347 and *P* = 0.179, respectively) (Fig. 2A–B). In contrast, differences between patients in the control and active groups according to the ARIA classification of allergic rhinitis types (i.e., intermittent and persistent) were statistically significant, showing lower frequency of persistent rhinitis in patients treated with AIT (*P* = 0.025) (Fig. 2C). However, the distribution of patients in the control and active treatment groups regarding the ARIA classification of rhinitis severity (i.e., mild, moderate, and severe) remained unchanged (*P* = 0.858) (Fig. 2D).

### Use of medication to treat allergic asthma and rhinitis

**Fig. 3:**
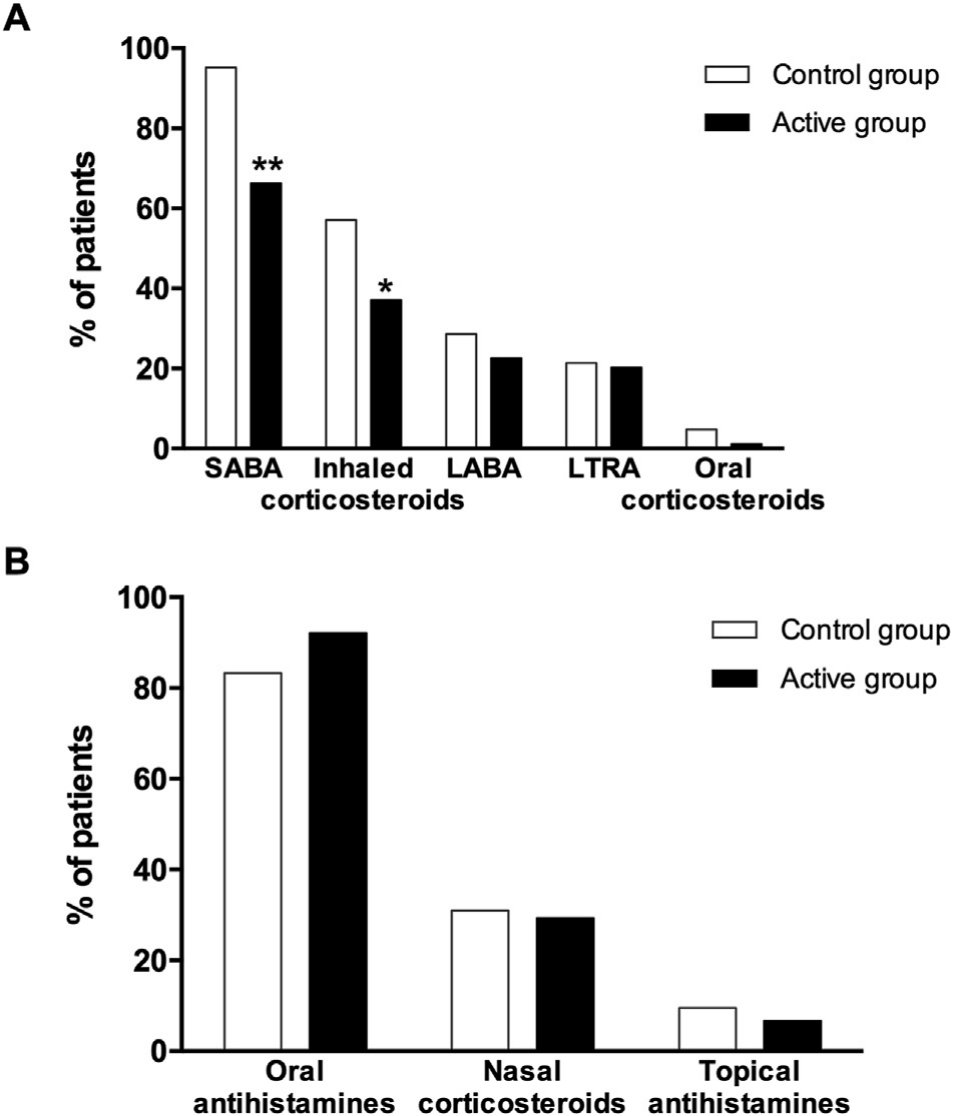
Use of medication to treat asthma (A) and rhinitis (B) in the active and control treatment groups. SABA, short-acting beta-agonist; LABA, long-acting beta-agonist; LTRA, leukotriene receptor antagonist. Chi-square test, ∗*P* < 0.05, ∗∗*P* < 0.01

The use of medication to treat allergic asthma decreased in patients treated with AIT compared with patients receiving conventional treatment. The frequency of patients using short-acting beta-agonists (SABAs) and inhaled corticosteroids was significantly lower in the active group (66.3% and 37.1%, respectively) compared to the control group (95.2% and 57.1%, respectively) (*P* = 0.001 and *P* = 0.031). Likewise, the use of long-acting beta-agonists (LABAs), leukotriene receptor antagonists (LTRAs), and oral corticosteroids tended to be lower in the active group, albeit differences lacked statistical significance (Fig. 3A). In contrast, the use of medication to treat allergic rhinitis, including nasal corticosteroids and oral and topical antihistamines, remained unchanged between control and active groups (Fig. 3B).

### Effectiveness outcomes according to treatment period and composition

Effectiveness outcomes, including unscheduled visits to a healthcare center and emergency room admissions (number of visits and percentage of patients), GEMA classification of asthma, and use of medication to treat asthma, were similar regardless of composition of Pollinex Quattro® and treatment period. Effectiveness variables were similar between patients treated with a Pollinex Quattro® composition of 100% olive pollen and 50% olive pollen/50% grass pollen (*P*; not significant for all variables) (Table 2). The profiles of sensitization to pollens in the two patient groups receiving the different AIT compositions are described in Table 3. Likewise, effectiveness variables remained similar regardless of the period of treatment administration (October–December and January–March) (*P*; not significant for all variables) (Table 4).

**Table 2:** Effectiveness variables according to treatment composition.

	100% olive pollen n = 43	50% olive pollen/50% grass pollen n = 46	*p*-value
Number of Unscheduled Visits to Healthcare Centers during the Pollen Season, *mean (SD)*	1.28 (1.30)	0.91 (1.19)	0.168^a^
Number of Unscheduled Visits to Emergency Rooms during the Pollen Season, *mean (SD)*	0.21 (0.71)	0.02 (0.15)	0.096^a^
Patients Visiting Healthcare Centers during the Pollen Season, %	69.8	56.5	0.196^b^
Patients Visiting Emergency Rooms during the Pollen Season, %	11.6	2.2	0.075^b^
GEMA Classification, %			
Intermittent	46.5	57.8	0.488^b^
Mild Persitent	32.6	31.1	
Moderate Persistent	18.6	11.1	
Severe Persistent	2.3	0	
Use of Medication to Treat Asthma, %			
Short-Acting Beta-Agonists	62.8	69.6	0.499^b^
Inhaled Corticosteroids	41.9	32.6	0.367^b^
Long-Acting Beta-Agonists	27.9	17.4	0.235^b^
Leukotriene Receptor Antagonist	18.6	21.7	0.713^b^
Oral Corticosteroids	2.3	0	0.483^c^

a Student's T-test.

b Chi-Square.

c Fisher Test

**Table 3:** Profiles of Pollen Sensitization according to Allergen Immunotherapy Composition, *n (%)*.

	100% Olive Pollen n = 43^a^	50% Olive Pollen/50%Grass Pollen n = 46^a^
Olive only	22 (51.2)	0 (0)
Olive and grass	2 (4.6)	15 (32.6)
Olive and other pollens	10 (23.2)	1 (2.2)
Olive, grass and other pollens	5 (11.6)	24 (52.2)

a Total number of patients per group. Data regarding sensitization to pollens other than olive and grass was unavailable for 4 and 6 patients in the 100% olive pollen and 50% olive pollen/50% grass pollen treatment composition groups, respectively

**Table 4:** Effectiveness variables according to treatment period.

	October–December n = 28	January–March n = 61	*p*-value
Number of Unscheduled Visits to Healthcare Centers during the Pollen Season, *mean (SD)*	1.46 (1.75)	0.92 (0.90)	0.129^a^
Number of Unscheduled Visits to Emergency Rooms during the Pollen Season, *mean (SD)*	0.11 (0.42)	0.11 (0.55)	0.948^a^
Patients Visiting Healthcare Centers during the Pollen Season, %	64.3	62.3	0.857^b^
Patients Visiting Emergency Rooms during the Pollen Season, %	7.1	6.6	0.919^b^
GEMA Classification, %			
Intermittent	53.6	51.7	0.513^c^
Mild Persitent	28.6	33.3	
Moderate Persistent	14.3	15	
Severe Persistent	3.6	0	
Use of Medication to Treat Asthma, %			
Short-Acting Beta-Agonists	57.1	70.5	0.216^b^
Inhaled Corticosteroids	28.6	41	0.260^b^
Long-Acting Beta-Agonists	10.7	27.9	0.072^b^
Leukotriene Receptor Antagonist	14.3	23	0.345^b^
Oral Corticosteroids	3.6	0	0.138^c^

a Student's T-test.

b Chi-Square.

c Fisher Test

### Safety outcomes

A total of 12 (13.5%) and two (2.2%) patients in the active group experienced 13 local and two systemic adverse reactions (ARs), respectively, none of which was serious. Systemic ARs included hypertensive crisis, which resolved in 20 min without needing any treatment, and low-grade fever. Local ARs included local edema (n = 9), injection site swelling (n = 2), erythema (n = 2), unspecified delayed local reaction (n = 1), pruritus (n = 1), local hives (n = 1), and heat (n = 1) (Table 5). Of the 12 patients who experienced local adverse reactions, three were children and adolescents; no systemic adverse reactions were reported in these age groups.

**Table 5:** Description of adverse reactions.

	n	Treatment
**Systemic reactions**	2	
Hypertensive crisis	1	Patient recovered in 20 min without treatment
Low-grade fever	1	500 mg paracetamol
**Local reactions**	13	
Swelling		
Local swelling at the injection site of diameter >10 cm	2	N/A
Edema		
diameter >10 cm	1	Local cold and Methylprednisolone aceponate (corticoid)
diameter 6 cm	1	Local cold and Fluticasone propionate (corticoid)
diameter 5 cm	1	N/A
with pruritus	1	N/A
with erythema	1	N/A
diameter 8–10 cm with heat	1	Local cold, oral Cetirizine (antihistamine) (10 mg) and oral prednisone (glucocorticoid). Resolved in 24 h.
Unspecified	3	N/A (n = 2) Local cold and prednicarbate (corticoid)
Erythema		
with local hives	1	N/A
Delayed local reaction of diameter 10 cm	1	Patient recovered without treatment

N/A not-available; information was requested but was unavailable

## Discussion

In this retrospective controlled study including patients with allergic asthma sensitized to olive pollen, with and without allergic rhinitis, we provided real-world evidence regarding the effectiveness and safety of Pollinex Quattro®. Patients treated with AIT experienced a low incidence of adverse reactions and experienced a lower number of unscheduled visits to the healthcare center and emergency room during the pollen season compared to patients receiving their usual pharmacological treatment. Differences in asthma classification revealed a trend towards improvement, albeit not statistically significant, with a relevant concomitant decrease in the use of SABAs and inhaled corticosteroids. Pollinex Quattro® showed similar effectiveness outcomes irrespective of the treatment period and composition.

Results from this study, showing that the out-of-season course of four Pollinex Quattro® injections improved allergic asthma during the pollen season, were obtained using different effectiveness variables collected from patients’ medical records. Asthma symptoms, measured using the GEMA classification, showed a trend towards a decrease in the active group, albeit not significant. AIT is contraindicated in patients with severe and uncontrolled asthma and therefore, patients receiving AIT lacked severe symptoms and had good asthma control, likely attenuating the observed effects of AIT. In contrast, the use of SABAs and inhaled corticosteroids to treat asthma symptoms significantly decreased in the active group, while rhinitis medication remained unchanged. According to the routine clinical practice in Spain, rhinitis medication is prescribed in advance for the entire pollen season and doses are not adjusted, unlike medication to treat asthma, which is gradually adjusted according to symptom control. The absence of dose adjustments according to rhinitis symptom control during the pollen season likely explain the lack of differences in use of rhinitis medication between active and control treatment groups. The remaining variables, including unscheduled visits to the healthcare center and emergency room, were, in contrast to the previous patient-reported variables, registered events and therefore objective and robust measures of treatment effectiveness. Both variables significantly decreased in the active vs. the control group, with a concomitant decrease in their associated odds ratio. To our knowledge, this is the first study in patients sensitized to olive pollen assessing unscheduled visits to the healthcare center and the emergency room, as previous studies assessed other immunological and clinical parameters.^29^, ^30^, ^31^ Nevertheless, similar to this study, previous trials, which were mostly prospective, concluded that subcutaneous immunotherapy (SCIT) provides a positive clinical benefit to patients with allergic rhinitis and asthma caused by olive pollen.^29^, ^30^, ^31^, ^32^, ^33^, ^34^, ^35^

Similarly to previous studies with Pollinex Quattro®, effectiveness outcomes remained unchanged regardless of the treatment period (October–December vs. January–March), allowing a flexible out-of-season administration throughout an extended period of time.^36^^,^^37^ Furthermore, the lack of influence of allergen composition (100% olive pollen vs. 50% olive pollen/50% grass pollen) on treatment effectiveness demonstrated that Pollinex Quattro® was effective in patients polysensitized to olive and grass pollen. In this regard, sensitization to olive and grass pollen are often found in the same patient and both species share pollination periods, between weeks 17 and 22 of the year (month of May).^6^^,^^14^^,^^38^ For these reasons, even though the focus of this study was to assess Pollinex Quattro® with an olive pollen extract (100% olive pollen), this study included patients receiving AIT additionally containing grass pollen extract (50% olive and 50% grass pollen), reflecting the epidemiology of these allergies.

The administration of Pollinex Quattro in only four out-of-season injections promotes treatment adherence and, despite the ultra-short course, this AIT showed a good effectiveness profile during the following pollen season. The unique formulation of Pollinex Quattro® in an allergoid by chemical modification with glutaraldehyde and combined with two different adjuvants, MCT and MPL, enables its ultra-short injection course. MCT slows the distribution of the allergoid after the subcutaneous injection and acts as a biodegradable adjuvant, while MPL stimulates the T_H_1 pathway of the unspecific immune response, enhancing the immunogenicity of the allergoid.^17^^,^^19^ In this respect, MCT and MPL have been shown to synergistically induce immune tolerance,^39^ likely explaining the good effectiveness profile of Pollinex Quattro®.

While specific guidelines for the use of AIT to treat allergic rhinitis symptoms have been developed, recommendations regarding prescription of AIT in patients with allergic asthma are still controversial, and the European Academy of Allergy and Clinical Immunology (EAACI) is still developing specific guidelines.^28^^,^^40^^,^^41^ However, reviews of previous clinical trials evaluating AIT to treat asthma due to different allergens concluded that AIT effectively reduced allergic asthma symptoms and use of medication.^28^^,^^42^^,^^43^ These studies reported increased risk of systemic adverse reactions associated with AIT in patients with allergic asthma, albeit at relatively low rates, and excluded AIT as an indication in patients with uncontrolled asthma, similar to the American Academy of Allergy, Asthma & Immunology (AAAAI).^15^^,^^44^^,^^45^ In our study, only two mild systemic adverse reactions were reported (2.2% rate), consisting in hypertensive crisis and low-grade fever, supporting the safety of the ultra-short injection course with Pollinex Quattro®.

Results from this study should be interpreted in the context of the limitations associated with retrospective studies, including missing data across variables and time points. In addition to the effect of missing values, the study was smaller than the estimated sample size, further decreasing the data available for analysis and its statistical power. Nevertheless, most of the comparisons yielded statistically significant results. This retrospective study was conducted in the real-world setting and consequently, the number of available variables was limited to those assessed and recorded in the routine practice. Additionally, due to its retrospective nature, daily symptoms and medication use were not registered, potentially resulting in an overestimation of treatment effects. Pollinex Quattro® is generally administered before three-to-five consecutive pollen seasons and therefore, despite its long-term efficacy in previous studies, longer studies are likely necessary in patients sensitized to olive pollen.^21^ Regardless of these limitations, the results from this study were obtained from real-world patients including children, adolescents, and adults, without strict selection criteria, and treated according to the routine clinical practice, likely mirroring results from the general population. Additionally, this was a multicenter study, probably reflecting the heterogeneity of patients from different regions. Furthermore, despite its retrospective design, our study included a control treatment group with demographic, clinical, and treatment characteristics similar to the active treatment group, enabling comparisons between patients evaluated during the same pollen seasons and therefore, most likely exposed to similar allergen concentrations. The results obtained from this retrospective real-world study warrant future prospective studies and clinical trials to obtain a comprehensive picture of the safety and effectiveness of Pollinex Quattro® in patients sensitized to olive pollen and polysensitized to olive and grass pollen.

## Conclusions

Our results obtained from patients with allergic asthma, with and without allergic rhinitis, show that AIT specific for olive or for grass and olive pollen is effective and safe in the real-world setting. Despite the short study duration, one course of treatment with Pollinex Quattro® resulted in less unscheduled visits to the healthcare center and emergency room and use of medication during the pollen season, with a low incidence of associated adverse reactions, suggesting that patients sensitized to olive pollen and polysensitized to olive and grass pollen may benefit from this treatment.

## Financial Support

This work was supported by Allergy Therapeutics Ibérica (Sant Joan Despí, Barcelona, Spain).

## Agreement to publish the work

All authors have reviewed and approved the final version of the manuscript.

## Authors’ contributions

All authors contributed to the conception and design of the study, or acquisition of data, or analysis and interpretation of data; all authors critically revised the article for important intellectual content and gave their final approval of the version to be submitted.

## Ethics approval and consent to participate

All included patients and legal representatives of patients <18 years signed a written informed consent before any data was recorded. The study was conducted in accordance with the Helsinki Declaration and the local Personal Data Protection Law (LOPD 15/1999); the study protocol was approved by the local Ethics Committee CEIM/CEI Provincial de (Granada, Spain).

## Editorial policy confirmation and agreement

I hereby state that the manuscript, including related data, figures and tables, has not been previously published and has not been submitted to any other journal (in any other language or any other type of publication), either by me or any of my co-authors.

## Availability of data and materials

The anonymized datasets collected during the current study are available from the corresponding author on reasonable request.

## Declaration of competing interest

The authors declare no conflict of interest.
